# Silicon: Potential to Promote Direct and Indirect Effects on Plant Defense Against Arthropod Pests in Agriculture

**DOI:** 10.3389/fpls.2016.00744

**Published:** 2016-06-13

**Authors:** Olivia L. Reynolds, Matthew P. Padula, Rensen Zeng, Geoff M. Gurr

**Affiliations:** ^1^Institute of Applied Ecology, Fujian Agriculture and Forestry University, Fuzhou, FujianChina; ^2^Graham Centre for Agricultural Innovation, New South Wales Department of Primary Industries, Menangle, NSWAustralia; ^3^Proteomics Core Facility, School of Life Sciences, University of Technology Sydney, Sydney, NSWAustralia; ^4^College of Life Science, Fujian Agriculture and Forestry University, Fuzhou, FujianChina; ^5^Graham Centre for Agricultural Innovation, Charles Sturt University, Orange, NSWAustralia

**Keywords:** herbivore, HIPV, effector proteins, insect–plant interactions, trophic interactions, resistance mechanisms, omics, systems biology

## Abstract

Silicon has generally not been considered essential for plant growth, although it is well recognized that many plants, particularly Poaceae, have substantial plant tissue concentrations of this element. Recently, however, the International Plant Nutrition Institute [IPNI] (2015), Georgia, USA has listed it as a “beneficial substance”. This reflects that numerous studies have now established that silicon may alleviate both biotic and abiotic stress. This paper explores the existing knowledge and recent advances in elucidating the role of silicon in plant defense against biotic stress, particularly against arthropod pests in agriculture and attraction of beneficial insects. Silicon confers resistance to herbivores via two described mechanisms: physical and biochemical/molecular. Until recently, studies have mainly centered on two trophic levels; the herbivore and plant. However, several studies now describe tri-trophic effects involving silicon that operate by attracting predators or parasitoids to plants under herbivore attack. Indeed, it has been demonstrated that silicon-treated, arthropod-attacked plants display increased attractiveness to natural enemies, an effect that was reflected in elevated biological control in the field. The reported relationships between soluble silicon and the jasmonic acid (JA) defense pathway, and JA and herbivore-induced plant volatiles (HIPVs) suggest that soluble silicon may enhance the production of HIPVs. Further, it is feasible that silicon uptake may affect protein expression (or modify proteins structurally) so that they can produce additional, or modify, the HIPV profile of plants. Ultimately, understanding silicon under plant ecological, physiological, biochemical, and molecular contexts will assist in fully elucidating the mechanisms behind silicon and plant response to biotic stress at both the bi- and tri-trophic levels.

## Introduction

### Silicon and the Soil

Silicon is the second most abundant element, after oxygen, in the Earth’s crust and in the soil solution (Epstein, 1994). It is mainly present in the soil solution in the form of silicic acid, H_4_SiO_4_, since this is the only form of water-soluble silicon. Soil concentrations typically range from 0.1 to 0.6 mM (Epstein, 1994). This concentration range is similar to that of major inorganic nutrients including potassium, calcium, and sulfate in the soil solution (Epstein, 1972). Several factors influence soil silicon availability to plants, including soil type, parent material, land use, organic matter, temperature, soil pH, and texture (Liang et al., 1994; Alexandre et al., 1997; Struyf et al., 2010; Cornelis et al., 2011; Han et al., 2011; Miles et al., 2014; Anda et al., 2015).

### Silicon and Plants

Silicon is taken up by plants via the transpiration stream (i.e., passive uptake) and is transported from the roots to the shoots as monosilicic acid, where it is deposited as solid, amorphous, hydrated plant silica (SiO_2_.*n*H_2_O; Jones and Handreck, 1967). Once deposited, silicon is not remobilized (Raven, 1983). Silicon is transported in the plant through the xylem via apoplastic transport (Raven, 1983) and must remain in solution (i.e., remain unpolymerized) during this passage; however the mechanisms preventing polymerization are not well understood (Epstein, 1994). Active silicon uptake is exhibited by some plant species including rice *Oryza sativa* L. (Takahashi et al., 1990; Henriet et al., 2006; Liang et al., 2006), as is rejective uptake (i.e., uptake at rates lower than passive; Takahashi et al., 1990). The existence of these processes indicates that, in some plant taxa at least, plant silicon levels are actively manipulated. Selection pressure for the evolution of active silicon uptake and metabolism is evident in the beneficial effects of silicon to plants under abiotic and biotic stress. However, silicon has not generally been recognized as an essential plant nutrient, though recently the International Plant Nutrition Institute [IPNI] (2015), Georgia, USA listed silicon as a “beneficial substance” (International Plant Nutrition Institute [IPNI], 2015).

The positive effects of silicon against abiotic and biotic stress are not always obvious since the extent of silicon accumulation differs among plant species and cultivars (Deren, 2001; Mitani and Ma, 2005; Keeping and Reynolds, 2009). Terrestrial plants have tissue concentrations of silicon, ranging from 1 to 15% dry weight (Epstein, 1994), with a very irregular distribution among the plant kingdom (Epstein, 1999). In agricultural systems, silicon is applied as a crop protection treatment and this is the major focus of this review. Major crops that respond to silicon application include some monocotyledonous plants such as rice, maize, *Zea mays* L., and wheat, *Triticum aestivum* L., that actively absorb and accumulate high amounts of silicon, together with some dicotyledonous crops such as cotton (*Gossypium hirsutum* L.), soybean [*Glycine max* (L.) Merr.], some vegetables (e.g., cucurbits) and fruit crops (e.g., tomato (*Lycopersicon esculentum* Mill.) that accumulate silicon through specific transporters (Liang et al., 2015). While it is well documented that sugarcane responds strongly to silicon fertilization, active absorption of silicon has not been demonstrated and an active transporter has not yet been found. More recently, high-throughput sequencing and easier access to genomic data has enabled accurate determination of the ability of a plant to accumulate silicon, based on its genetic predisposition (Liang et al., 2015).

Until the discovery of specific genes involved in silicon uptake, silicon accumulation in plants was little understood. These silicon transporter genes, influx and efflux (LSi1 and LSi2, respectively), responsible for silicon uptake by the roots were first described in rice (Ma et al., 2006, 2007). Homologs are now reported in barley, *Hordeum vulgare* L., maize, and wheat (Chiba et al., 2009; Mitani et al., 2009a,b; Montpetit et al., 2012), with pumpkin, *Cucurbita moschata*, Poir. the first dicot to record a gene encoding a silicon influx transporter, LSi1 (Mitani et al., 2011) and two efflux transporters, CmLSi2-1 and CmLSi2-2 (Mitani-Ueno et al., 2011) followed by two putative influx silicon transporter genes (GmNIP2-1 and GmNIP2-2) in soybean (Deshmukh et al., 2013) and cucumber (CSiT-1, CSiT-2; Wang et al., 2015). An influx transporter has also been identified in the primitive plant, horsetail, *Equisetum arvense* L. (Gŕegoire et al., 2012). A silicon influx transporter, LSi6, present in the root tips, leaf sheaths and leaf blades has also been identified in several graminaceous species, including rice, and is responsible for xylem unloading of silicon (Yamaji et al., 2008).

### Silicon and Stress

The beneficial effects of silicon application on plant growth and crop yield are well documented (for a recent review see Guntzer et al., 2012), but it is in the mitigation of both abiotic and biotic plant stresses, where the application of silicon demonstrates its real potential (Keeping and Reynolds, 2009). Notably, biochemical or molecular responses (and frequently growth/yield responses) due to silicon fertilization, are usually not apparent unless in the presence of a biotic (or abiotic) stressor. Studies have shown resistance to a range of abiotic stress factors including drought and salinity stress, heavy metal toxicity, excess nitrogen and phosphorous, and lodging (for a recent review see Liang et al., 2015). Biotic stressors may come in the form of plant pathogens, including fungi, bacteria, viruses, and animals (vertebrate and arthropod herbivores). Defense against biotic stress, has centered around two main mechanisms, mechanical (physical), and biochemical or molecular.

There is a dominance of work on fungal pathogens, compared with other disease-causing agents. Those fungal pathogens defined as biotrophic or hemibiotrophic, including the powdery mildews and blast fungus (*Magnaporthe grisea* (T.T. Hebert) M.E. Barr), appear to be better controlled by silicon than are necrotrophs (Liang et al., 2015). The reasons for this are increasingly becoming apparent, with a recent study showing that while silicon contributes to *Arabidopsis* defense priming following pathogen infection, that silicon will confer protection even when priming is altered, indicating other mechanisms may be involved (Vivancos et al., 2015). Evidence suggests that silicon may interfere with effector proteins released by these pathogens, permitting the plant to mount better defense reactions (Vivancos et al., 2015). Other work has confirmed the role of silicon in priming plants in plant–pathogen interactions (Fauteux et al., 2005; Chain et al., 2009; Van Bockhaven et al., 2013). It is thought that the work on silicon and effector proteins may assist in developing a unifying theory around the mode of action of silicon in alleviating biotic stresses (Vivancos et al., 2015). A recent, comprehensive review of silicon and plant–pathogen interactions in agriculture is provided by Liang et al. (2015).

Vertebrate herbivores are probably the least studied biotic stressors, against which silicon provides some protection, and research in this area has largely focused on natural ecological systems. We briefly review this field because it has some relevance to arthropod pests given that plant defenses are at the heart of the phenomenon. The majority of studies have been on field voles, *Microtus agrestis* L (Rodentia: Cricetidae), showing reductions in the body weight and growth rate of juveniles and adults when fed on silicon-treated grasses (Massey and Hartley, 2006; Massey et al., 2008). Recent laboratory work demonstrated that grasses employ several defense strategies against *M. agrestis* including silicon, endophytes, and secondary metabolites (Huitu et al., 2014). It is hypothesized that induction of silicon-based plant defense in response to herbivore damage may influence rodent population cycles (Massey et al., 2008). In sites where *M. agrestis* population density was high, silica levels in the leaves of their food plant, collected several months later were also high and vole populations afterward declined, while population density increased where vole population density was initially low and silicon levels were also low (Massey et al., 2008). A key food species, *Deschampsia cespitosa* L., of *M. agrestis* exhibits a delayed defensive response to grazing by increasing silica concentrations (Reynolds et al., 2012). Further, the authors presented theoretical modeling that predicts that this response alone could lead to population cycles observed in *M. agrestis* and in other graminivorous rodent populations, where populations that reach sufficiently high densities can induce silica defenses in their food source.

Studies on the root vole, *Microtus oeconomus* (Pallas, 1776), have shown that changes in the silicon content of tussock sedges may be induced by variations in vole population densities (Wieczorek et al., 2015). However, no correlation was shown between the silicon content in the faeces of *M. oeconomus* and survival rate (Wieczorek et al., 2015). A very recent study in Poland demonstrated that the amount of silica in plants, fed upon by voles, leaves a traceable record in their dental microwear textures, and that these differ through different phases of vole population cycles (Calandra et al., 2016). The authors hypothesize that the high quantity of phytoliths, produced due to intense grazing in peak years, can result in malocclusion and other dental abnormalities, and may explain how these silicon-based plant defenses contribute to population crashes. Silicon-treated wheat plants showed enhanced resistance to feeding by the wild rabbit (*Oryctolagus cuniculus* L.), a major vertebrate pest of cereals in the United Kingdom (Cotterill et al., 2007). Further, severe, potentially lethal feeding damage due to rabbit browsing, was reduced in silicon-treated wheat by over 50%. Feeding preference in sheep (*Ovis aries* L.), in response to silicon availability, did not differ within a grass species; however, there were differences in the bite rate and feeding preference between grass species, with these differences more obvious in silicon-treated plants (Massey et al., 2009). Further, silicon influenced grass preference less in palatable species, compared to less desirable species, an effect that appeared to be due to the most palatable species containing relatively little silicon even after supplementation, and being less tough (Massey et al., 2009).

Numerous studies have shown enhanced resistance of plants treated (soil and/or foliar application) with silicon to insect herbivores and other arthropods, including folivores (Korndorfer et al., 2004; Redmond and Potter, 2006; Massey et al., 2007; Han et al., 2015), borers (Kvedaras and Keeping, 2007; Kvedaras et al., 2007a,b, 2009; Hou and Han, 2010; Keeping et al., 2013; Vilela et al., 2014), phloem (Correa et al., 2005; Goussain et al., 2005; He et al., 2015) and xylem feeders (Yoshihara et al., 1979), mites (Nikpay and Nejadian, 2014) and nematodes (Silva et al., 2015). However, there is no consistent evidence for silicon having a greater effect in any particular feeding guild or taxon (Keeping and Kvedaras, 2008). The vast majority of studies are at two trophic levels, with few studies at the third trophic level (Reynolds et al., 2009; Gurr and Kvedaras, 2010; Kvedaras et al., 2010). A comprehensive review of earlier work on the role of silicon against herbivorous insects was provided by Reynolds et al. (2009).

This paper explores the more recent advances in the role of silicon in ameliorating the effects of biotic stress, particularly that caused by arthropods from agricultural systems, and the response of their natural enemies, together with the mechanisms involved in bi- and tri-trophic interactions. We also review literature relating to the effects of silicon on plant pathogens where this helps illustrate underlying mechanisms of plant defense that may have relevance to arthropods. Understanding the role and function of silicon against arthropod pests, will ultimately enable us to optimize the use of this element in the context of sustainable agriculture.

## Bi-Trophic Interactions

Silicon fertilization of plants has proven to be effective in controlling insect herbivores and other arthropods. Indeed, silicon application has become a routine practice in rice production in some countries, including Japan, where a silicon fertilizer was first applied to any crop worldwide (Ma and Takahashi, 2002). In agricultural systems, silicon is typically applied to the soil, or as a foliar spray to the vegetation. It is feasible that foliar application of silicon can have an effect on arthropods, e.g., via surface pH or osmotic effects. However, there is now considerable evidence, notably in fungal systems, that soil applied silicon leads to significantly more silicon accumulation in plant tissues, than foliar applications and produces much better results against biotic stressors (Liang et al., 2005, 2015; Guével et al., 2007; Dallagnol et al., 2015). Details of the mechanisms underlying silicon-mediated plant resistance against biotic stress are increasingly becoming clear, with an increase in the number of publications in this area in recent years.

### Physical Mechanisms

An increased physical barrier produced by silicon deposition beneath leaf cuticles has long been considered to represent a major component underlying silicon-mediated plant resistance to insect pests. Silicon deposition contributes to increased rigidity and abrasiveness of plant tissues, thereby forming a mechanical barrier and reducing their palatability and digestibility to both vertebrate (Massey and Hartley, 2006, 2009) and invertebrate herbivores (Goussain et al., 2005; Kvedaras et al., 2007a; Massey and Hartley, 2009). Increased abrasiveness of leaves due to silicon deposition reduces food quality for herbivores and may cause wear of herbivore mouthparts, which further reduces feeding efficiency and growth rates (Massey and Hartley, 2009). Conversely, using a simple method to determine mandibular wear (Smith et al., 2007), it was shown that although there was a trend for increased wear in *Eldana saccharina* larvae that developed on silicon-treated sugarcane, the ability of larvae to renew their mandibles at each moult probably allows them to compensate for increased wear (Kvedaras et al., 2009). Finely ground wollastonite (CaSiO_3_) in artificial diets at rates of up to 3.3% silicon had no significant effect on larval growth of *Helicoverpa armigera* (Hübner; Lepidoptera: Noctuidae) and *Helicoverpa punctigera* Wallengren, suggesting that silicon may not be directly deleterious to insects via ingestion and other mechanisms may be involved in silicon-mediated plant resistance (Stanley et al., 2014). It should be noted, however, that by grinding the silicon, this has likely removed potential abrasive attributes, in addition to the potential effects of soluble-silicon-induced plant defenses.

Using energy-dispersive X-ray (EDX) and X-ray mapping, it was shown that the pattern of silicon deposition in sugarcane, especially at the internode and root band, is likely the reason (at least, in part) for enhanced resistance of silicon-treated sugarcane to penetration and feeding by *E. saccharina* at these sites (Keeping et al., 2009). Further, epidermal silicon was higher in the control (i.e., no silicon treatment), *E. saccharina* resistant cultivar, than the susceptible control cultivar, suggesting that such differences in silicon-mediated resistance exist to a large extent due to the varying ability of cultivars to deposit silicon within the stalk epidermis (Keeping et al., 2009), thus preventing *E. saccharina* penetration (Kvedaras and Keeping, 2007). A more recent study using scanning electron microscopy and EDX compared four grass species, and showed that spine and phytolith morphology both within and between species may be more important than leaf silicon concentration in determining the abrasiveness and/or digestibility of leaves and thus the effectiveness of anti-herbivore defense (Hartley et al., 2015). The authors showed that all the grasses tested were able to deposit new types of silicon-based structures when silicon supply was increased. These changes were particularly evident when the leaves were mechanically damaged; however, damage in the absence of additional silicon did not produce such structures (Hartley et al., 2015).

### Biochemical/Molecular Mechanisms

**FIGURE 1:**
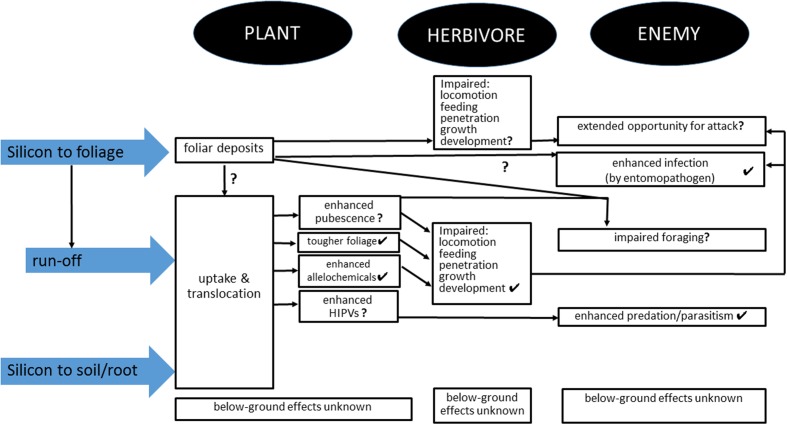
**Summary of mechanisms by which application of silicon treatments to plants may affect the plant, herbivores and natural enemies.** Ticks indicate empirically supported effects and question marks indicate untested effects. See text for details.

McNaughton and Tarrants (1983) were the first to show induction of silica. They showed that plants growing in a more heavily mammal-grazed grassland in the Serengeti, Tanzania, accumulated more silica in their leaf blades relative to plants from a less heavily grazed site, and blade silica content was higher when plants were defoliated, suggesting that silicification is an inducible defense against mammalian herbivores. Massey et al. (2007) demonstrated in a laboratory study, that feeding by both a mammal, *M. agrestis* and an insect, *Schistocerca gregaria* Forskal (Orthoptera: Acrididae) led to increased levels of silica in grass leaves. Other recent studies on arthropods have demonstrated that silicon-mediated anti-herbivore defense is both inducible and allelochemical-mediated (Gomes et al., 2005; Kvedaras et al., 2010; Costa et al., 2011) and these effects can complement the physical effects described above, leading to impaired feeding, growth, and development (**Figure 1**).

Increasing evidence shows that silicon treatment increases transcript levels of defense-related genes, thereby enhancing the activities of plant defensive enzymes (Liang et al., 2003; Cai et al., 2008; Rahman et al., 2015) leading to increased accumulation of defensive compounds, such as phenolics, phytoalexins, and momilactones (Fawe et al., 1998; Rodrigues et al., 2004; Rémus-Borel et al., 2005). Gomes et al. (2005) showed that the addition of silicon strongly enhanced wheat resistance to greenbug *Schizaphis graminum* (Rondani; Hemiptera: Aphididae). Further, silicon pre-treatment increased the activities of the defensive enzymes peroxidase, polyphenoloxidase, and phenylalanine ammonia lyase. In particular, silicon facilitated the strongest resistance if wheat plants had previously been infested with aphids. Chérif et al. (1994) found that silicon-treated cucumber plants show increased activity of the enzymes peroxidase, polyphenoloxidase, β-1,3 glucanase, and chitinase in response to infection by pathogens. Perennial ryegrass (*Lolium perenne* L.) grown in silicon-amended soil exhibited greater activity of peroxidase and polyphenoloxidase, higher levels of several phenolic acids, including chlorogenic acid and flavonoids, and enhanced expression levels of genes encoding phenylalanine ammonia lyase (PALa and PALb) and lipoxygenase (LOXa) in response to infection by *Magnaporthe oryzae* (T.T. Hebert) M.E. Barr (Rahman et al., 2015). Histological and ultrastructural analyses revealed that silicon mediates active localized cell defenses, and epidermal cells of silicon-treated plants displayed specific defense reactions including papilla formation, production of callose, and accumulation of glycosilated phenolics in response to pathogen infection by the fungus *Blumeria graminis* f. sp. *tritici* (DC.) Speer (Bélanger et al., 2003). Silicon-mediated brown spot resistance in rice plants is independent of the classic immune hormones, salicylic acid and jasmonic acid (JA; Van Bockhaven et al., 2015). Conversely, silicon mounted rice resistance to the brown spot fungus *Cochliobolus miyabeanus* (Ito and Kuribayashi) Dastur, by interfering with the production and/or action of fungal ethylene, prevents the fungus from suppressing the rice innate immune system (Van Bockhaven et al., 2015).

Pre-treatment with certain chemicals or previous biotic stressor may provoke a specific physiological state in plants called “priming” (Fauteux et al., 2006; Hao et al., 2012; Worrall et al., 2012; Aimé et al., 2013). Primed plants are thus physiologically prepared to induce quicker and/or stronger defense responses upon subsequent attack, providing plants with a more effective means to respond to challenges (Ton et al., 2006; Jung et al., 2009; Slaughter et al., 2012; Ye et al., 2013). A recent study demonstrates that silicon is able to prime jasmonate-mediated defense responses and rice defense against a chewing herbivore, the rice leaffolder, *Cnaphalocrocis medinalis* (Lepidoptera: Pyralidae; (Ye et al., 2013). More interestingly, activation of jasmonate signaling in turn promotes silicon accumulation in rice leaves, indicating a strong interaction between silicon and jasmonate in rice defense against insect herbivores. Some recent studies have shown that silicon can also prime plants for alleviating biotic stress imposed by pathogens (Ghareeb et al., 2011; Rahman et al., 2015). Vivancos et al. (2015) showed that priming is also an important mechanism of silicon-mediated resistance of *Arabidopsis thaliana* (L.) Heynh. against powdery mildew caused by *Golovinomyces cichoracearum* (DC.). Further, this work has also revealed that silicon may interfere with effector proteins released by such biotrophic pathogens, suggesting that mechanisms other than salicylic acid-dependent plant defense priming are involved (Vivancos et al., 2015). It has been suggested that priming of plant defense responses, alterations in phytohormone homeostasis, and interaction with defense signaling components are all potential mechanisms involved in regulating silicon-triggered resistance responses (Van Bockhaven et al., 2013). Silicon has also been demonstrated to prime plants for resistance against abiotic stresses (Ahmed et al., 2013). Research on silicon-mediated herbivore resistance lags far behind that on silicon-mediated disease resistance. Further studies are needed to determine the exact nature of silicon-primed anti-herbivore defense and indeed other mechanisms that may play a role in plant resistance to biotic stressors. For example, effectors that modulate plant defenses have also been identified in the saliva of insects (for a review see Hogenhout and Bos, 2011) and it is feasible that a similar mechanism proposed for plant pathogens, also operates for insects, although this remains to be elucidated.

Recent developments regarding the understanding of molecular mechanisms controlling silicon accumulation and the discovery of silicon transporters have enabled a ready ability to classify a plant as Si-competent, or not. This will enable a better understanding of the role of silicon in several fundamental aspects of ecology concerning plant fitness under stress (Deshmukh and Bélanger, 2015).

## Tri-Trophic Interactions

Natural enemies of herbivores can be important in the management of agricultural pest species. Evidence for this includes the wide literature on biological control using predators, parasitoids and entomopathogens. In this section we consider what is currently the least thoroughly investigated aspect of plant–silicon–herbivore interactions: the mechanisms by which the application of silicon compounds may affect the impact of natural enemies on herbivores.

### Entomopathogenic Microorganisms

Entomopathogens are increasingly used in arthropod pest management. However, as this approach uses applications of live organisms rather than chemicals, as in conventional insecticide use, particular attention needs to be given to maximizing the viability and impact of the treatment on the target pest. In work with the fungus *Beauveria bassiana* (Bals.-Criv.) Vuill., 1912, potassium silicate was added to nutrient solutions applied to plant roots seven days after inoculation with spider mite, *Tetranychus urticae* Koch (Gatarayiha et al., 2010). Potassium silicate alone did not kill the pest mites, but when used at the higher rates, equivalent to 80 and 160 mg of pure silicon per liter, pest mortality caused by *B. bassiana* was up to 92%. The authors of that study hypothesized that silicon application primed biochemical defenses in the plants (see above) which interfered with the feeding of mites making them more susceptible to the entomopathogen (**Figure 1**).

### Predators

Of particular relevance to the possible effects of silicon on non-entomopathogenic natural enemies is a study of induced defense in rice (Ye et al., 2013). This study, employing rice mutant lines in which genes for jasmonate synthesis or jasmonate perception were silenced, showed a strong interaction between soil-applied silicon and JA in defense against insect herbivores. This involved priming of JA-mediated defense responses by silicon and the promotion of silicon accumulation by JA (Ye et al., 2013). While that work did not extend to considering natural enemies it is significant for third trophic level effects because it identified a relationship between silicon and JA. Silicon is translocated within plants in the form of monosilicic acid, Si(OH)_4_ which is reported as an elicitor for systemic stress signals including JA (Fauteux et al., 2005). JA, in turn, is the primary signaling pathway that is activated by chewing herbivores leading to herbivore-induced plant volatiles (HIPV) production (Dicke et al., 1999, 2009).

**FIGURE 2:**
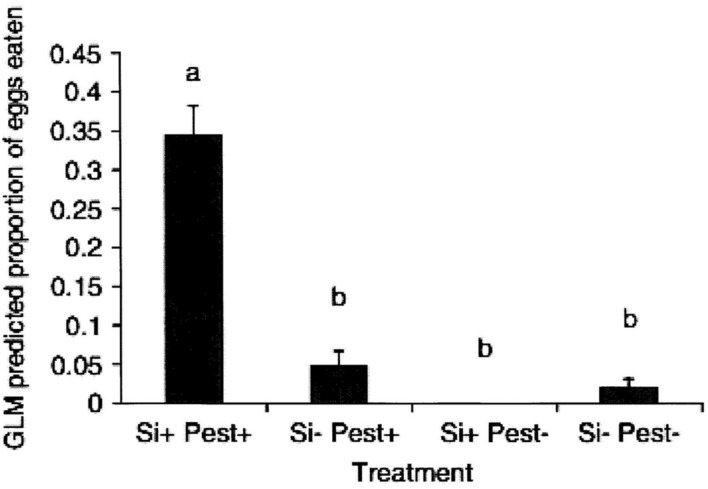
**The effect of prior treatment with potassium silicate (silicon+) and infestation with 10 *H. armigera* larvae/plant (pest+) on the proportion of prey eggs removed from potted cucumber plants over a 24-h period when exposed to predators in the field.** (*N* = 4), columns with differing letters differ (LSD test, *P* = 0.05). (Reproduced with permission from Kvedaras et al., 2010).

The first published study of the effects of silicon on plant defense in which HIPV-mediated effects has been the focus was in cucumber (Kvedaras et al., 2010). That work demonstrated that soil-applied silicon enhanced the attraction of the predator *Dicranolaius bellulus* (Guerin-Meneville; Coleoptera: Melyridae) to *Helicoverpa armigera* (Hubner; Lepidoptera: Noctuidae) infested cucumber plants in a Y-tube olfactometer bioassay. Further, a small-scale field trial, using *H. armigera* eggs affixed to potted cucumber plants, before they were placed in a field plot of lucerne, showed that increased biological control by “wild” predators was significantly higher for soil-applied, silicon-treated plants than for control plants (Kvedaras et al., 2010; **Figure 2**). The authors hypothesized that this was due to a change in the plant volatile profile (HIPVs) produced by cucumber plants when attacked by an herbivore. Additional studies to measure and identify the compounds produced by pest-infested silicon-treated and untreated cucumber plants are worthwhile. Similar work on grapevines has yielded preliminary evidence for volatile-mediated defenses to promote predator attraction to pest-infested plants (Connick, 2011). A study of the volatiles produced by grapevines infested by the Lepidoptera pest, grapevine moth *Phalaenoides glycinae* (Lewin; Lepidoptera: Noctuidae) found that soil applied potassium silicate had profound effects. Seven volatile compounds emitted from *P. glycinae*-infested grapevines were identified and *n*-heptadecane found to be produced in significant amounts only by silicon-treated plants. Cis-thio rose oxide production, in contrast, was significantly lower in silicon-treated grapevines. A second study in that thesis found that the attractiveness of grapevines infested with the lightbrown apple moth (*Epiphyas postvittana* (Walker; Lepidoptera: Tortricidae) was positively correlated with plant foliar tissue concentration of silicon (Connick, 2011).

The impact of natural enemies on herbivores may be enhanced by mechanisms other than induced, indirect defenses based on HIPVs. By extending development time, and particularly the period over which neonate larvae feed on the exterior of plants before being able to penetrate the plant cuticle and commence mining or boring, herbivores are exposed to a higher risk of attack by predators. Delayed penetration was evident in a study of sugarcane borer, *E. saccharina* (Kvedaras and Keeping, 2007). Massey and Hartley (2006) reported similar findings for *Spodoptera exempta* Walker feeding on grass with high silicon levels. Many natural enemies forage for prey by locomotion over the foliar surface, so the practice of applying silicon treatments to the above ground plant parts could have physical or chemical effects on natural enemy foraging (**Figure 1**). Examples of recent studies that included treatments with foliar applied silicon are Dalastra et al. (2011) and de Assis et al. (2012, 2013), and in the latter of those studies, there was no effect of foliar treatments to potato plants on predatory beetles, although the plants treated with silicic acid were less preferred by defoliators. Further work needs to test for the strength of such effects on a wider range of natural enemy taxa.

Foraging of predators may also be affected by foliar pubescence, especially glandular trichomes. The latter produce irritant, toxic and adhesive liquid secretions from the tips that can provide high levels of protection from foliar-associated herbivores (Gurr and McGrath, 2002) but can also affect natural enemies (Simmons and Gurr, 2004, 2005). When subject to herbivores, plants have the capacity to regenerate new leaves that exhibit enhanced densities of trichomes, an induced defense that is under the control of JA (Yoshida et al., 2009). This form of induced defense is remarkable in taking place over days rather than the timespan of hours as in the case of induced production of semiochemical volatiles. This phenomenon has relevance to the interplay between silicon and plant defense because plant-available silicon influences the JA signaling pathway (Ye et al., 2013). Accordingly, the phenomenon of herbivore-attacked plants producing more hirsute foliage is another form of plant defense that we hypothesize may by amplified by silicon pre-treatment (**Figure 1**).

Not only might plant-available silicon promote the density of trichomes on young foliage, work on deposition patterns of silica in the leaf epidermis suggests that the bases of trichomes is a major site in cucumber (Samuels et al., 1991a,b), while in the grasses *D. cespitosa* and *Festuca ovina* L., silica was particularly evident in the tips of spines under control conditions, but was distributed throughout the spine and the leaf surface when silicon fertilized (Hartley et al., 2015). The epicarp hairs present on the mature caryopses of the four cereals, barley, oats, rye, and wheat (Bennett and Parry, 1981) are also important silicon deposition sites, particularly in the tips of hairs where it is most likely to promote adverse effects on herbivores including – potentially – human consumers of grain products (Parry et al., 1984). It remains to be tested whether the potentially adverse effects of trichomes on predators are exacerbated by silicon supplementation and the extent to which any such effects are offset by stronger effects on herbivores.

Among studies of the effects of silicon on pests that do consider third trophic level effects, these tend to use designs that are not well suited to detecting the full range of possible mechanisms that may operate. An example is work by Moraes et al. (2004), with the lacewing *Chrysoperla externa* Steinmann in which wheat aphid (*Schizaphis graminum* (Rondani; Hemiptera: Aphididae) prey were removed from the test plants before being exposed to the predators. Since predators were not exposed to plants or their volatiles, they would have been unable to detect HIPV-mediated effects, though effects related to prey quality could be assessed.

A major limit on our current understanding of the effects of silicon on natural enemies is the apparent absence of studies on below-ground effects. Many arthropod pests cause important damage to plant roots so studies of how silicon might promote natural enemies such as predacious beetle larvae and entomopathogenic nematodes would be valuable.

### Parasitoids

Of the three types of natural enemies, parasitoids are the least well studied in relation to plant available silicon, though many of the comments made above, for established and possible effects on predators (**Figure 1**), will apply to parasitoids. Of particular significance is the wealth of evidence for HIPVs attracting parasitoids to pest-infested plants (Dicke et al., 2009). The only study with silicon-treated and un-treated plants in which a parasitoid was considered is that by Moraes et al. (2004) with *Aphidius colemani* Viereck (Hymenoptera: Aphididae). Unfortunately, this confined wasps to narrowly spaced wheat plants and, because it used non-choice conditions, would not have allowed HIPV-mediated effects to be apparent.

## How “Omics” Support Plant Defense Studies?

**FIGURE 3:**
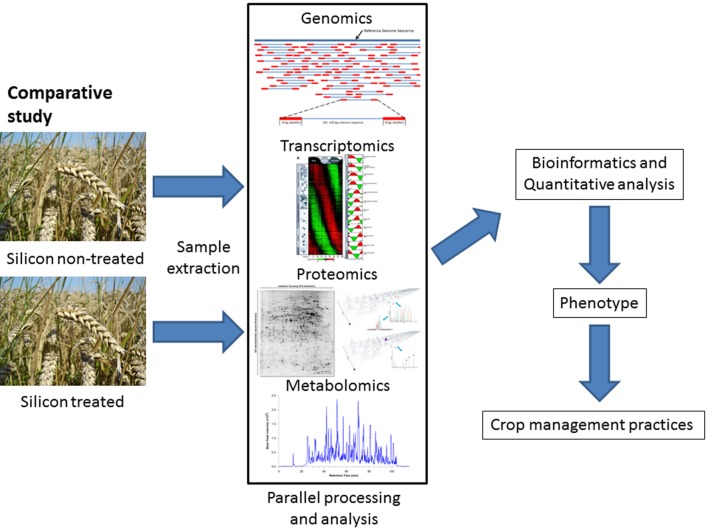
**The workflow for the application of -omics technology to quantify phenotypic changes in plants due to silicon treatment.** In a comparative study, parallel samples are grown under laboratory conditions or in the field with one subject to silicon treatment. After the application of appropriate sample extraction techniques to obtain mRNA, proteins or metabolites in an unbiased and comprehensive manner, the samples are subjected to parallel analysis to obtain a comprehensive dataset of the transcriptome, proteome, and metabolome. These datasets are then analyzed in bioinformatic pipelines to identify the components and quantify the differences in abundance of specific mRNAs, proteins or metabolites, which can then be related to phenotypic changes in the plant, such as resistance to a herbivore or pathogen. This information can then be utilized in crop management practices. A similar analysis could be applied to an ecological system, in order to understand the role of silicon (whether naturally occurring or supplemented) in ecological processes, for example comparing grazed versus ungrazed pastures.

To understand how the addition of silicon to a plant’s environment can improve plant defense, the plant as a whole must be considered through global analysis of the major responsive components of the DNA, RNA, proteins, and metabolites which are then holistically viewed using bioinformatics (**Figure 3**).

While system-wide analysis has long been applied to plants, their application to analyzing plant defense has been limited (Chen et al., 2005; Giri et al., 2006; Thivierge et al., 2010; Lewandowska-Gnatowska et al., 2011; Duceppe et al., 2012; Timbo et al., 2014) and analyzing silicon’s role even more so. Numerous reductionist experiments targeting specific proteins or enzymes have shown that silicon treatment induces plant defensive enzymes (Liang et al., 2003; Cai et al., 2008), leading to the accumulation of defensive compounds and metabolites (Fawe et al., 1998; Rodrigues et al., 2004).

But the power of -omics approaches lies in its non-targeted nature, allowing the unearthing of unexpected changes. Transcriptome analysis represents the only -omic analysis of silicon’s effects, with a study on challenged *A. thaliana* showing silicon treatment causes a decrease in primary metabolism that allows a more efficient defense response (Fauteux et al., 2006). A similar analysis was also conducted on rice (Ye et al., 2013), as indicated above. Recent work has sought to establish the “Prime-ome”, or the mechanism behind how a plant defends itself or is in a “primed state” to rapidly respond to attack by insects and microbial pathogens (Balmer et al., 2015). Not surprisingly, the available -omics scale data shows that the plant’s response depends on the priming inducer and the pathogen, which is also observed in defense against arthropods (Balmer et al., 2015). Silicon’s role in defense against herbivores remains vastly understudied by -omics methodologies which would reveal the role of, as yet, untargeted molecules, including proteins and metabolites, through global analysis.

Transcriptomics alone is insufficient to understand an organism’s phenotype (Barah and Bones, 2015) as it is the proteome and metabolome that provide the molecular mechanisms that allow a plant to defend itself (Oliveira et al., 2014). While proteomics and metabolomics are rapidly maturing fields, they are still limited by the issues of throughput and the depth of proteome and metabolome coverage due to the dynamic range of concentration of the molecules present (Jorge et al., 2015). The abundance of proteins can vary by 7–10 orders of magnitude (Ly and Wasinger, 2008; Zubarev, 2013) and the existence of a proteoform is often reported by the detection of only a single peptide (Mallick et al., 2007). Without an equivalent of PCR utilized in genomics and transcriptomics, the only way to reliably detect and quantify the abundance of low copy number proteins is to start with more material (Zubarev, 2013) and fractionate the proteins to isolate those of high abundance from the rest (Stasyk and Huber, 2004; Righetti et al., 2005; Ly and Wasinger, 2011). The same logic applies to metabolites but in both cases the number of fractions requiring analysis increases.

In the case of proteomics, fractionation of intact proteins reduces this increase compared to “shotgun” peptide-centric methods while retaining the option of utilizing 2D-PAGE as a further fractionation and quantification method (Coorssen and Yergey, 2015). To determine plant defense responses as a result of silicon treatment, 2D-PAGE has the distinct advantage of quantifying protein abundance changes prior to identification. This is contrary to LC/MS/MS methodologies where identification of peptides and their assignment to a protein isoform needs to be performed prior to quantitation. Thus, 2D-PAGE can decrease the number of samples requiring analyses by mass spectrometry (MS), freeing valuable instrument time. In proteomics, the issue of throughput is being addressed somewhat by faster instrument scan speeds (Richards et al., 2015), the adoption of ultra high-pressure chromatography (Kocher et al., 2011; Thakur et al., 2011) and data-independent acquisition (DIA) techniques in LC/MS/MS (Huang et al., 2015). DIA methodologies have also been applied to measure nitrogen flux and metabolism (Ullmann-Zeunert et al., 2012) indicating that DIA could have application in quantitative metabolomics, in order to assess how changes in the levels of specific metabolites can be related to observed plant defensive phenotypes.

## Conclusions and Future Directions

There is now considerable literature supporting the role of silicon as a physical defense mechanism, and a growing number of published works on the role of silicon-mediated biochemical defense. However, there are few references on the role of silicon in tri-trophic interactions.

Research should focus on understanding the relative importance of both physical and biochemical defence and how (if) this differs between herbivores. A meta-analysis of the literature would be valuable to discern if silicon has a greater effect in certain feeding guilds or taxons. Understanding the interaction between silicon and the plant defense pathways, and if there is a similar mechanism acting against insects, and pathogens, will also be paramount, as there is a wealth of literature on silicon/pathogen interactions that can inform arthropod work.

Future researchers need to address the lack of knowledge on below-ground effects of silicon application to plants on predators. There is a more general dearth of knowledge on how silicon might alter root toughness and chemical defenses. There is also a need to test for the effects of foliar deposits from foliar applied silicon on natural enemy foraging and impact. Work also needs to consider the possibility that changing the plant surface, by denser or more robust trichomes, may have negative effects on natural enemy foraging (**Figure 1**). More generally, workers need to consider the effects of silicon under field conditions (something done quite extensively for mammals in natural ecological systems) and be less reliant on greenhouse and laboratory studies, especially those that make it impossible for natural enemy mediated effects on herbivores to be apparent. Finally, there are currently no published studies of the effects of silicon on HIPV production but such work is known to be underway. If strong evidence is forthcoming for effects on the blend of HIPVs, this will add impetus to the need for greater attention to be given to the third trophic level in studies of silicon on plant defenses.

Using system-wide analysis or -omics technologies would permit us to not only understand silicon’s role in the production of defense-related compounds, but in the production of HIPVs, in addition to the associated energy costs to the plant. This could potentially inform the manipulation of plants to minimize herbivory and maximize the impact of natural enemies.

Modern approaches of transcriptomics, proteomics, metabolomics, and transgenic mutants will serve as powerful tools for dissecting the underlying mechanism/s involved in silicon and plant defense. In an era when sustainable pest management is receiving more attention than ever before, due largely to restrictions or the withdrawal of toxic pesticides, because of their negative impacts on human and environmental health, silicon treatment should be more widely considered and tested as a pest management option.

## Author Contributions

OR and GG developed the concept, drafted, and critically revised the manuscript. MP and RZ drafted and critically revised the manuscript.

## Conflict of Interest Statement

The authors declare that the research was conducted in the absence of any commercial or financial relationships that could be construed as a potential conflict of interest. The reviewer JD and handling Editor declared their shared affiliation, and the handling Editor states that the process nevertheless met the standards of a fair and objective review.
